# Supplementary material for: Mammals from ‘down under’: a multi-gene species-level phylogeny of marsupial mammals (Mammalia, Metatheria)
Source: PeerJ. 2015 Feb 26;3:e805. doi: 10.7717/peerj.805 (PMC4349131; doi:10.7717/peerj.805)
Supplement: Table S2 [file peerj-03-805-s002.doc]

P A U P *

Portable version 4.0b10 for Microsoft Windows 95/NT

Fri Aug 30 09:57:47 2013

-----------------------------NOTICE-----------------------------

This is a beta-test version. Please report any crashes,

apparent calculation errors, or other anomalous results.

There are no restrictions on publication of results obtained

with this version, but you should check the WWW site

frequently for bug announcements and/or updated versions.

See the README file on the distribution media for details.

----------------------------------------------------------------

paup> paup> exe Matrixsimple.nex;

Processing of file "Matrixsimple.nex" begins...

Data read in DNA format

Data matrix has 251 taxa, 18723 characters

Valid character-state symbols: ACGT

Missing data identified by '?'

Gaps identified by '-'

"Equate" macros in effect:

R,r ==> {AG}

Y,y ==> {CT}

M,m ==> {AC}

K,k ==> {GT}

S,s ==> {CG}

W,w ==> {AT}

H,h ==> {ACT}

B,b ==> {CGT}

V,v ==> {ACG}

D,d ==> {AGT}

N,n ==> {ACGT}

4 trees read from TREES block

Time used = 0.01 sec

Rooted tree(s) input but current criterion and/or option settings would specify

unrooted trees. Do you want to "deroot" the tree(s)? (Y/n) paup> n

Processing of file "Matrixsimple.nex" completed.

paup> paup> lscores all/nst=6 basefreq=estimate rmatrix=estimate rates=gamma shape=estimate pinvar=estimate shtest=rell;

Likelihood scores of tree(s) in memory:

Likelihood settings:

Number of substitution types = 6

Substitution rate-matrix parameters estimated via ML

Nucleotide frequencies estimated via ML

Among-site rate variation:

Assumed proportion of invariable sites = estimated

Distribution of rates at variable sites = gamma (discrete approximation)

Shape parameter (alpha) = estimated

Number of rate categories = 4

Representation of average rate for each category = mean

These settings correspond to the GTR+G+I model

Number of distinct data patterns under this model = 13391

Molecular clock not enforced

Starting branch lengths obtained using Rogers-Swofford approximation method

Branch-length optimization = one-dimensional Newton-Raphson with pass

limit=20, delta=1e-006

-ln L (unconstrained) = unavailable due to missing-data and/or ambiguities

Tree 1 2 3 4

--------------------------------------------------------------

-ln L 435351.24761 435357.08824 435353.47676 435361.16372

Base frequencies:

A 0.356962 0.357002 0.356987 0.357060

C 0.256672 0.256711 0.256641 0.256582

G 0.147662 0.147553 0.147661 0.147621

T 0.238705 0.238733 0.238711 0.238737

Rate matrix R:

AC 1.36138 1.36030 1.36176 1.36191

AG 4.64617 4.64949 4.64626 4.64637

AT 1.64413 1.64206 1.64314 1.64210

CG 1.04780 1.04776 1.04666 1.04785

CT 10.47159 10.46090 10.47016 10.47065

GT 1.00000 1.00000 1.00000 1.00000

P_inv 0.185240 0.185531 0.185415 0.185642

Shape 0.507463 0.507746 0.507721 0.508060

Time used to compute likelihoods = 10:45:41.9

Shimodaira-Hasegawa test:

SH test using RELL bootstrap (one-tailed test)

Number of bootstrap replicates = 1000

Tree -ln L Diff -ln L P

---------------------------------------------

1 435351.24761 (best)

2 435357.08824 5.84062 0.463

3 435353.47676 2.22915 0.725

4 435361.16372 9.91611 0.263

paup> paup> exit

paup> paup> quit;
